# Supplementary material for: Association between oral microbiome and five types of respiratory infections: a two-sample Mendelian randomization study in east Asian population
Source: Front Microbiol. 2024 Apr 10;15:1392473. doi: 10.3389/fmicb.2024.1392473 (PMC11039966; doi:10.3389/fmicb.2024.1392473)
Supplement: Supplementary file 1 [file Data_Sheet_1.zip › Supplementary Materials/MR plots of saliva/Chronic sinusitis/s__Gemella_haemolysans_B_mgs_2903/scatter.pdf]

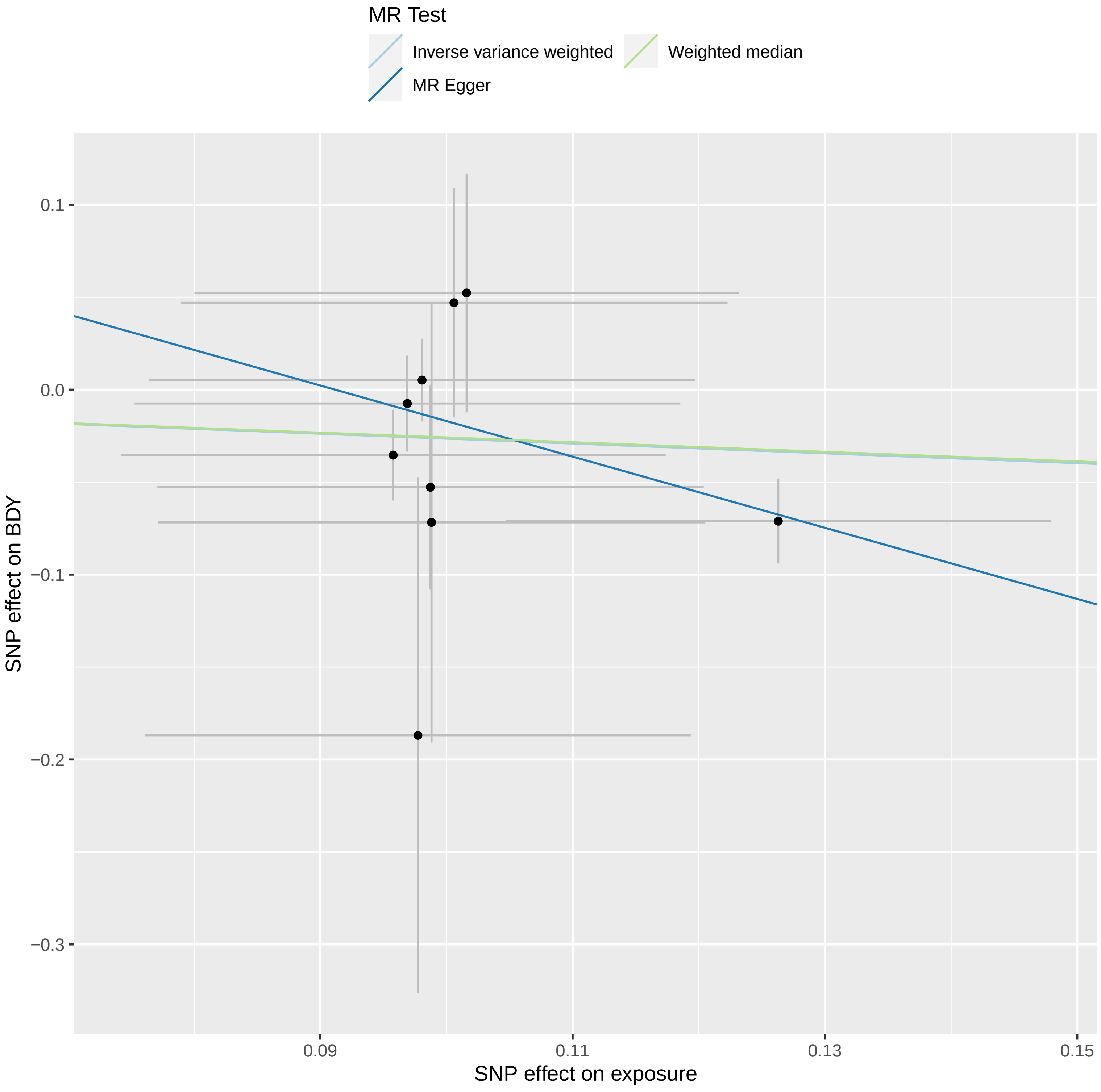
MR Test
Inverse variance weighted
Weighted median
MR Egger
0.1
0.0
−0.1
−0.2
−0.3
SNP effect on BDY
0.09
0.11
0.13
0.15
SNP effect on exposure
